# Anti-IL-6 and PD-L1 antibody combination therapy reduces tumor progression in murine models of pancreatic cancer

**DOI:** 10.1186/2051-1426-3-S2-P366

**Published:** 2015-11-04

**Authors:** Thomas Mace, Reena Shakya, Jason Pitarresi, Shannon Loftus, Benjamin Swanson, Gregory Young, Xioling Zhong, Teresa Zimmers, Michael Ostrowski, Thomas Ludwig, Mark Bloomston, Tanios Bekaii-Saab, Gregory Lesinski

**Affiliations:** 1 Department of Internal Medicine, James Comprehensive Cancer Center, The Ohio State University, Columbus, OH, USA; 2 The Ohio State University, Columbus, OH, USA; 3 Center for Biostatistics, The Ohio State University, Columbus, OH, USA; 4 Indiana University Simon Cancer Center, Indianapolis, IN, USA; 5 Department of Internal Medicine, James Comprehensive Cancer Center, The Ohio State University, Columbus, OH, USA

## 

Checkpoint inhibition has gained traction as an immunotherapeutic approach. However, limited efficacy has been observed in patients with pancreatic ductal adenocarcinoma (PDAC). Prior studies by our group have indicated that human pancreatic stellate cells (PSC), a major component of the pancreatic stroma secrete copious amounts of IL-6, which can act through the Jak/STAT pathway to promote immunosuppression. We hypothesized that inhibition of IL-6 would enhance the efficacy of anti-PD-L1 checkpoint inhibitor therapy in preclinical models of PDAC. In support of our hypothesis are histologic data confirming stromal regions in human PDAC tumors stain strongly positive for IL-6, as opposed to areas of ductal carcinoma. Consistent with prior published studies, PD-L1 expression was also detectable in patient PDAC patient samples. Nanostring analysis of RNA isolated from patient-derived PSC exhibit prominent expression of cytokines and chemokines including IL-6, CCL11, and FGF7 as compared to RNA isolated from normal human pancreatic fibroblasts. *In vivo* studies were conducted to assess the efficacy of anti-PD-L1 when combined with anti-IL-6 antibody (Ab) in mice bearing subcutaneous syngeneic, Panc02 tumors. These data revealed significant inhibition of tumor growth in mice treated with the combination as compared to mice treated with isotype control Ab or either Ab alone (n=6; p < 0.02). Further analysis revealed a greater percentage of CD4^+^ and CD8^+^ effector T lymphocytes (CD62L^-^CD44^-^) in tumors from combination-treated mice as compared to isotype controls (p < 0.04). This treatment combination was also evaluated in an aggressive genetically engineered PDAC model (Kras^LSL-G12D^, Trp53^LSL-R270H^, Pdx1-cre, Brca2^F/F^ (KPC-Brca2 mice)). This model has many characteristics that better recapitulate features of human PDAC, including high tumoral PD-L1, stromal IL-6 production, nuclear pSTAT3 expression in the tumor microenvironment and systemic immune suppression. Treatment of KPC-Brca2 mice with anti-IL-6 and PD-L1 antibodies three times weekly for 2 weeks limited tumor progression. This was supported by histologic analysis confirming a marked shift in the proportion of pancreata with PanIN1/2 lesions rather than PanIN3 or adenocarcinoma foci in combination treated mice as compared to mice receiving isotype or either Ab as a single agent alone (n=5, p < 0.05). Further, analyses revealed significantly increased intratumoral CD3+ T lymphocytes (p < 0.001) and a greater percentage of splenic Th1 cells (CD4^+^CXCR3^+^CXCR4^-^CCR6^-^) in the combination group as compared to isotype controls (p < 0.05). These results indicate that targeting of IL-6 may enhance the efficacy of anti-PD-L1 in PDAC.

